# Sinus membrane thickness of healthy endodontically treated maxillary molars

**DOI:** 10.1007/s00784-024-05815-y

**Published:** 2024-07-08

**Authors:** Deniz Yanık, Ahmet Mert Nalbantoğlu, Kürşat Er

**Affiliations:** 1 Faculty of Dentistry, Department of Endodontics, Süleyman Demirel University, Isparta, Türkiye; 2 Faculty of Dentistry, Department of Periodontology, Süleyman Demirel University, Isparta, Türkiye; 3 Faculty of Dentistry, Department of Endodontics, Akdeniz University, Antalya, Türkiye

**Keywords:** apical foramen, endodontic treatment, maxillary molar, maxillary sinus, sinus membrane thickness

## Abstract

**Objective:**

The study aimed to investigate the sinus membrane thickness (SMT) adjacent to healthy endodontically-treated maxillary molars with or without protruded apical foramen into the sinus cavity using cone-beam computed tomography (CBCT).

**Materials and methods:**

Images of 207 non-smoker patients aged 18–40 were retrospectively analyzed, 140 were endodontically treated, and 136 were without endodontic treatment. Patients with any sinus pathology, teeth that have symptoms, or poor root filling were excluded. Study groups consisted of Group EM-I (endodontically treated and protruded apical foramen), Group EM-C (endodontically treated and contacted apical foramen), and similarly without endodontic treatment; Group M-I and Group M-C. SMT upon the mesial, distal, and palatal roots was measured. One-way ANOVA and Student’s t-tests were performed.

**Results:**

Group EM-I had the thickest sinus membrane compared to other groups (*p* = 0.013). SMT values were 2.37–2.60 mm in Group EM-I, and 1.34–1.58 mm in other groups. Thickening (> 2 mm) percentages were 33.45% in Group EM-I and between 4.25 and 8.25% in other groups. No statistical difference was detected between first and second molars and genders (*p* > 0.05).

**Conclusion:**

When the apical foramen protruded into the sinus cavity, the conventional root canal treatment caused a minimal (between 2.37 mm and 2.60 mm) sinus membrane thickening with a rate of 33.45% based upon CBCT examinations.

## Introduction

The maxillary sinus is a pyramidal-shaped cavity located adjacent to the nasal cavity, maxillary posterior teeth, and orbit. The maxillary sinus membrane, which is named the Schnederian membrane, covers all maxillary sinus cavities. Maxillary sinus membrane thickness (SMT), which is approximately 1–2 mm under normal conditions, may altered by infection [1]. SMT thicker than 2 mm is a finding of the sinus infection [2]. One of the main reasons for the thickening of the sinus membrane is odontogenic infections. Spread of an endodontic pathology to the maxillary sinus, also called endo-antral syndrome, may prolong the treatment and worsen the clinical status [1–3]. According to the literature, approximately 50% of maxillary sinusitis is derived from an odontogenic pathology such as apical or marginal periodontitis [2, 3]. Especially, infection of maxillary molars tends to spread surrounding maxillofacial structures including the maxillary sinus due to the bone morphology and volume of the maxilla. The bone of the sinus floor adjacent to the apexes of the maxillary molar is evidently thin. In some cases, no bone exists between the root apices and the maxillary sinus, or the roots may even protrude into the sinus cavity. This type of anatomic relationship may result in maxillary sinus infections of endodontic origin.

Cone beam computed tomography (CBCT) is a crucial radiographic modality that allows a three-dimensional examination of both teeth and surrounding orofacial structures [4, 5]. Under low radiation dosage conditions, it provides a detailed image of maxillofacial regions and is very valuable and well-established in every branch of dentistry [4, 6]. The maxillary sinus is one of the well-observable anatomic structures via CBCT. CBCT, a 3D imaging modality, has superiority over traditional 3D modalities such as periapical radiography, in examining the relationship of maxillary posterior teeth with the sinus [4]. Previously, several studies examined the effect of primer or seconder endodontic infection, periapical lesion, and bone loss on the sinus using CBCT [1, 2, 7–10].

Considering the relationship between the root canal system established through the apical foramen and the surrounding tissues, it is necessary to ask the question: Is only the presence of an infection necessary for the thickening of the sinus membrane to occur? Or does the presence of endodontic treatment itself create a trauma effect on the sinus membrane? Therefore, the aim of the study is to evaluate the thickness of the maxillary sinus membrane related to endodontically treated maxillary molars. The null hypotheses of the study were established as follows (i) the presence of root canal treatment would not cause sinus membrane thickening and (ii) the presence of the root in the sinus would not cause sinus thickening.

## Materials and methods

### Protocol of the study

This retrospective study’s protocol was confirmed by the local ethics committee for human research (protocol number: 724). The protocol and design were determined in accordance with the guidelines of the Declaration of Helsinki, version 2008, and the guidelines were pursued in all stages of the study. To detect the minimum sample size, a power analysis was performed with 0.25 effect size, α errs 0.05, and power of 0.90 by the software of G*Power 3.1 (Heinrich–Heine–Universität, Düsseldorf, Germany). Power analysis showed the requirement of minimum sample size was a total of 232.

### Selection of samples

For the study, 300 CBCT images belonging to non-smoking patients who resorted to the private dental clinic between January 2017 and August 2021 were retrospectively scanned. All patients were native Turkish. Patients whose endodontic treatment was performed by one general dentist in the same clinic were selected. CBCT examinations were obtained owing to different indications including surgical extraction, implant placement, the examination of bone pathologies or temporomandibular joint, etc., and were not related to the present study. CBCT images were obtained using Orthophos (Sirona Dental Systems, Bensheim, Germany) with imaging parameters set as 85 kVp, 6 mA, 14.1 sn exposure time, 0.2 mm voxel size, and 8 × 4 cm field of view by the same technician according to the “as low as reasonably achievable” (ALARA) principle. Due to the nature of the retrospective study, the parameters of the images included in the study were all confirmed to be the same before analysis.

### Inclusion criteria

The inclusion criteria were CBCT images with endodontically treated maxillary molars of systematically healthy patients. Only patients between the ages of 18 and 40 were included [9]. Maxillary molars with periodontal health were selected. Periodontal health was determined as no interproximal bone loss of more than 2 mm. The included CBCT images belonged to vital teeth whose procedures were completed with single-visit root-canal treatment and composite filling. Conventional root canal treatments were performed with irrigation with routine sodium hypochlorite (2.52%) and ethylenediaminetetraacetic acid (17%), and using obturation materials of epoxy resin sealer and gutta-percha. It was taken into consideration that the adjacent teeth (mesially and distally) of the included samples were intact and healthy (no missing, no implant, no lesion, no decay, or filling). Only maxillary molars that contacted the maxillary sinus or whose roots extended into the sinus were measured (roots with bone in between the sinus cavity were not included). For the inclusion of the sample in the study, the apical foramen of the sample was required to be in contact with the sinus cavity. CBCT images of patients whose root canal treatments were performed in the same private clinic in 12–24 months before imaging were selected.

### Exclusion criteria

The exclusion criteria were smoking patients with nasal septum deviation, mucous retention cysts, any chronic or allergic sinus pathology, and jaw fracture. Patients with sinusitis and any sinus surgery history were excluded. Teeth in which the lateral surface of the root was in contact with the sinus cavity and the apical foramen remained within the borders of the maxillary bone were not included. Endodontically treated maxillary molars, those with dental implants in the adjacent tooth, those with periapical lesions, those with inadequate canal fillings, and those whose roots were in contact with the wisdom tooth were excluded. Adequate filling was described as a root canal filling that terminated at 0–2 mm from the radiographic apex [11]. The periapical lesion was determined according to the periapical index score [12]. Those whose PAI score was except 1 were excluded. Other endodontic procedures such as retreatment, vital pulp therapy, or root-end resection were excluded from the study. Teeth with infra or supra positions or rotated were excluded. In healthy maxillary molar groups, teeth with filling, caries, periapical lesions, any prosthesis, eroded crowns, and crowns without structural integrity were not included. For all groups, vertical root fractures, crown fractures, molars without antagonist teeth, and horizontal or vertical bone loss were excluded.

All inclusion and exclusion criteria were considered for both male and female subjects and for both first and second maxillary molars.

### Study groups

Maxillary molar teeth were divided into subgroups according to the presence of endodontic treatment and sinus relationships of the roots;

#### Group EM-I (endodontically treated molar - inside)

Endodontically treated maxillary molar with the protruded apical foramina into the sinus cavity.

#### Group EM-C (endodontically treated molar - contact)

Endodontically treated maxillary molar with apical foramina that come into contact with the sinus cavity.

#### Group M-I (molar without endodontic treatment - inside)

Maxillary molar without endodontic treatment with the protruded apical foramina into the sinus cavity.

#### Group M-C (molar without endodontic treatment - contact)

Maxillary molar without endodontic treatment with apical foramina that come into contact with the sinus cavity.

In the study, the patients whose root canal treatment was completed within 12–24 months were selected as the sample. To ensure homogeneity between groups and avoid confounders, this long period was examined by dividing it into 4 different time periods; time period I; 13–15 months, time period II; 16–18 months; time period III; 19–21 months; time period IV; 22–24 months. Analysis of the measurements was also carried out on the basis of these four time groups.

### Measurements of CBCT images

**Fig. 1:**
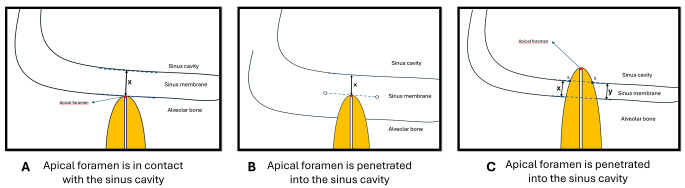
Schematic demonstration of measurement. Measurements (**A**) when the apical foramen contacts with the sinus cavity, (**B**) when the apical foramen penetrates the sinus cavity and the full thickness of the membrane is superior to the apex, (**C**) when the apical foramen penetrates the sinus cavity and the full thickness of the membrane, is inferior to the apex

**Fig. 2:**
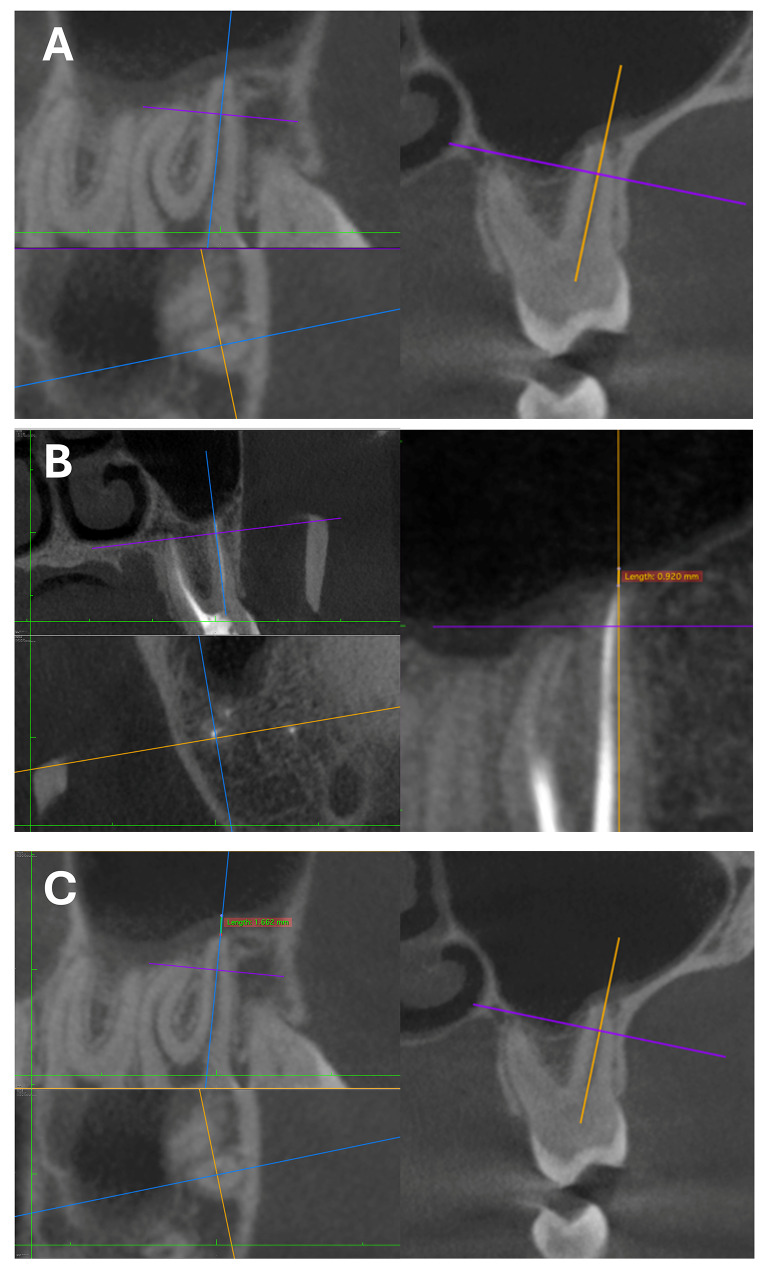
Examinations in axial, sagittal, and coronal sections. (**A**) Axial guided navigation method (**B**) SMT measurements of endodontically treated tooth (**B**) SMT measurements of tooth without endodontic treatment (SMT: sinus membrane thickness)

Before measurements, two observers (D.Y., a 7-year experienced endodontist, and A.M.N., a 12-year experienced periodontist) were calibrated. An oral and maxillofacial radiologist (independent observer, a 7-year experience) was consulted for the analysis of the images before calibration of two observers. For calibration, 30 images (approximately %10 of the total sample size) were evaluated, and the kappa score was stated (range from 0.85 to 0.91). Images were examined in coronal, axial, and sagittal planes. While making measurements, firstly the apical foramen was determined by the axial guided navigation method [13] in the sagittal section. In CBCT images of teeth whose apical foramen was in contact with the sinus, the SMT at the alignment of the apical foramen was measured from the shortest distance (Fig. 1, A). When the apical foramen protruded into the sinus and the aspect of the sinus membrane that faced the cavity was positioned at the upper of the apical foramen, the distance between the sinus floor and the surface of the sinus membrane facing the cavity was measured (Fig. 1, B). When the sinus membrane was positioned coronal to the apical foramen, the points where the root intersects the sinus membrane were determined distally (a) and mesially (b). A line was passed through points a and b. Another line parallel to this line was created on the surface of the sinus floor facing the cavity. The distance between two parallel lines was measured (Fig. 1, C). The mesial, distal, and palatal root was measured individually. Measurements were performed in sagittal sections at x5 magnification (Fig. 2).

Two observers of the study examined the images. The inter-class and intra-class reliability was analyzed with the measurements of two observers independently. If a disagreement about the interpretation of the samples occurred, the relevant sample would be excluded from the study. All distances were measured two times, and the average values were recorded for the statistical analysis. The measurements of 5 samples were measured at one time and a break was made. The adjustment of the optical visualization including the contrast or brightness of the image by the software tool was achieved.

### Statistical analysis

Statistical analysis was performed using SPSS version 26.0 for Windows (IBM Corp., Armonk, NY, USA). Kolmogorov-Smirnov and Levene’s tests were used to examine the normality and homogeneity of the study data. Student’s t-test and one-way ANOVA were performed for the statistical analysis. We evaluated the differences in the sinus membrane thickness between males and females, between first and second molars, and between the right and left sides. Interclass correlation coefficient (ICC) was carried out for the reliability of the observer. For the Student’s t-test and two-way ANOVA tests, the statistical significance was *p* < 0.05 with a 95% confidence interval, while for the ICC, the statistical significance level was *p* < 0.001.

## Results

After the exclusion criteria, a total of 276 maxillary molars (140 were endodontically treated, 136 were maxillary molars without endodontic treatment) belonging to 207 patients were analyzed. Table 1 demonstrates the average SMT values (in millimeters) of the study groups. SMT did not differ between the first and second molars (*p* > 0.05). Besides, according to the one-way ANOVA, SMTs of mesial, distal, and palatal roots were similar in the first and second molars (*p* > 0.05). SMTs of study groups were statistically different (*p* < 0.05). According to the post hoc Tukey test, the SMT of Group EM-I had the highest values (*p* = 0.013). However, no statistical difference was detected between Group EM-C, Group M-I, and Group M-C (*p* > 0.05).

**Table 1:** Sinus membrane thicknesses (in mm) of mesial, distal, and palatal canals of endodontically treated and healthy maxillary molars

		Total			
			First molar	Second molar	*P* value*
**Group EM-I** **(** ***n*** **= 67)**	Mesial	2.60 (± 0.23)^a^	2.57 (± 0.21)^a^	2.63 (± 0.24)^a^	*0.08*
	Distal	2.39 (± 0.25)^x^	2.71 (± 0.13)^x^	2.07 (± 0.32)^x^	*0.21*
	Palatal	2.37 (± 0.19)^m^	2.67 (± 0.17)^m^	2.07 (± 0.32)^m^	*0.07*
	*P value* ^*¥*^	*0.32*	*0.45*	*0.39*	
**Group EM-C** **(** ***n*** **= 73)**	Mesial	1.42 (± 0.17)^b^	1.23 (± 0.15)^b^	1.61 (± 0.22)^b^	*0.16*
	Distal	1.58 (± 0.28)^y^	1.74 (± 0.31)^y^	1.43 (± 0.26)^y^	*0.06*
	Palatal	1.34 (± 0.21)^n^	1.46 (± 0.17)^n^	1.23 (± 0.35)^n^	*0.25*
	*P value* ^*¥*^	*0.14*	*0.06*	*0.14*	
**Group M-I** **(** ***n*** **= 65)**	Mesial	1.48 (± 0.19)^b^	1.43 (± 0.13)^b^	1.53 (± 0.31)^b^	*0.34*
	Distal	1.36 (± 0.2)^y^	1.39 (± 0.22)^y^	1.34(± 0.19)^y^	*0.09*
	Palatal	1.41 (± 0.25)^n^	1.54 (± 0.32)^n^	1.29 (± 0.27)^n^	*0.32*
	*P value* ^*¥*^	*0.06*	*0.18*	*0.23*	
**Group M-C** **(** ***n*** **= 71)**	Mesial	1.47 (± 0.21)^b^	1.43 (± 0.18)^b^	1.51 (± 0.37)^b^	*0.13*
	Distal	1.53 (± 0.16)^y^	1.37 (± 0.16)^y^	1.69 (± 0.19)^y^	*0.42*
	Palatal	1.55 (± 0.28)^n^	1.63 (± 0.25)^n^	1.47 (± 0.33)^n^	*0.06*
	*P value* ^¥^	*0.08*	*0.52*	*0.34*	
*P* value^¥¥^		***0.013***	***0.001***	***0.001***	

In the column, for mesial (a, b), distal (x, y), and palatal canals (m, n) separately, different low superscript letters mean statistical difference according to one-way ANOVA (*p* < 0.05)

* means the comparison between first and second molars

^*¥*^ means the comparison between mesial, distal, and palatal canals

^¥¥^ means the comparison between groups

**Table 1:** Sinus membrane thickening (> 2 mm) percentages (%) of mesial, distal, and palatal roots of endodontically treated and healthy maxillary molars

	Total	First molar	Second molar
**Group EM-I** **(** ***n*** **= 75)**	33.45%	34.6%	32.3%
**Group EM-C** **(** *n* **= 84)**	4.25%	2.5%	6%
**Group M-I** **(** ***n*** **= 79)**	4.60%	3.43%	5.78%
**Group M-C** **(** ***n*** **= 87)**	8.25%	9%	7.5%

According to the Student’s t-test, there was no statistically significant difference between left and right molars in terms of the SMT (*p* > 0.05). In addition to this, no statistical difference was detected between males and females (*p* > 0.05).

In the study, there were 41 samples in time period I (18 in Group EM-I, and 24 in Group EM-C), 19 samples in time period II (8 in Group EM-I, and 11 in Group EM-C), 34 samples in time period III (14 in Group EM-I, and 24 in Group EM-C), and 46 samples in time period IV (28 in Group EM-I, and 18 in Group EM-C). There was no statistical difference between the time groups in Group EM-I and Group EM-C according to one-way ANOVA (*p* > 0.05).

The ICCs were excellent for the measurement of the SMTs in every study group (ICC = 0.817 for Group EM-I, ICC = 0.903 for Group EM-C, ICC = 0.867 for Group M-I, ICC = 0.912 for Group M-C).

## Discussion

According to our results, the most important result of the research is that if the apical foramen of an endodontically treated tooth penetrates the sinus, it may cause a thickening of the sinus membrane > 2 mm at a rate of 33.45%. Therefore, the null hypotheses of the study were partially accepted. Previously, the influence of maxillary teeth with primary or secondary endodontic infection, or periapical lesion on sinus membrane thickness was examined [2, 4, 5, 8, 14, 15]. However, there was no clear data about the effect of healthy endodontically treated teeth without any radiographic or clinical symptoms on sinus membrane thickening. van den Munckhof et al. measured SMT before and after root canal treatment [1]. This study examined the teeth requiring root canal treatment with or without periapical pathology and changes in SMT with root canal treatment. No clear data about the effect of root position and the relationship between apical foramina and the sinus cavity [1, 16]. Similarly, the effect of the presence of root canal filling, regardless of the position of the apical foramen and the quality of filling was reported [7, 16]. In the present study, the SMT adjacent to the healthy endodontically treated teeth with no pathology was analyzed, and it was reported that endodontic treatments performed to roots protruding into the sinus may cause thickening of the sinus membrane, even if there is no pathology.

In the present study, samples included teeth with adequate root canal fillings. Previous studies examining canal filling quality showed that the sinus membrane was thicker in teeth with poor filling quality [8, 10]. However, there is no detailed information about the root position, the presence of periapical lesions on teeth with inadequate filling, or single versus multiple visits. In this study, samples that were treated in a single visit, had adequate root canal filling, and had an apical foramen opening into the sinus were examined. According to the results of our study, the presence of root canal treatment caused thickening of the sinus when the root extended into the sinus.

In the present study, the effect of endodontically treated teeth with healthy periapical tissues on SMT was examined. During the study, the location of the apical foramen was reckoned with to understand the microtrauma effects of endodontic procedures. The endodontic treatment itself may affect the sinus physiology, especially when the apical foramen come into contact with the cavity because of the procedures including thermomechanical preparation or filling. Due to the apical foramen representing a space, it is too far from being an obstacle to the effects of endodontic procedures on the surrounding anatomical formations [17]. Therefore, even if endodontic treatment does not cause an infection in the tooth or surrounding tissues and is performed in accordance with standards in the treatment guidelines, the nature of the treatment may cause changes in the sinus membrane. During endodontic treatment, agents that may have an irritant or corrosive effect on healthy tissues are used for the elimination of bacteria in the root canal system [18]. Besides the root canal system is obturated with materials that are relatively not biocompatible, even if with a minimal adverse effect, compared to the biological properties of the pulp tissue itself [19]. That’s why every endodontic procedure is terminated coronal to the minor apical foramen and determining the root canal length is crucial in endodontics. However, at the point where the root canal filling ends, there is no hard tissue barrier between the obturation materials and living tissues, and the root canal obturation interacts with the vital tissues that have blood circulation through the apical foramen. Considering all these factors, it is necessary to ask the question: Is only the presence of an infection necessary for the thickening of the sinus membrane to occur? Or does the presence of endodontic treatment itself create a trauma effect on the sinus membrane?

In addition to odontogenic or nasal pathologies, SMT may vary owing to age, gender, and smoking habits. Previously, it was reported that the SMT got thicker with age and in males [2, 4, 14, 20]. Alghofaily et al. [8] reported no difference between age < 40 or > 40. On the other hand, according to the literature, the sinus membrane was getting thicker in the population with the age > 60 [9, 14, 20]. In our study, individuals between the ages of 18 and 40 were only included, so the analysis based on age groups was not performed. No difference was detected between genders, which confirmed the previous reports [8, 21, 22], however, contrary to others that report higher values in males [7, 20]. Further studies with higher sample sizes are needed to understand the combined effect of anatomic relationship and age.

The fact that the floor of the sinus cavity is not a flat surface when analyzed on 3D perspective, requires confirmation of measurements in two sections. Therefore, in this study, the data were examined in both coronal and sagittal sections. Previous studies have also conducted their examinations in more than one cross-Sects [1, 2]. In a previous study examining the periapical lesion in the tooth, both cross-sectional images were evaluated and a 93% correlation between the two images was reported [23]. In the present study, there is no difference between sections. In studies examining sinus membrane thickening or root relationships, which sections are examined may affect the results. In a study where the periapical lesion was evaluated on CBCT, the correlation between two different sections was reported as 93% [23].

The normal thickness of the sinus membrane, which consists of ciliated respiratory epithelium, has been reported to be between 0.80 and 1.99 mm on average [7]. Sinus membrane thickening is one of the most important indicators of sinus pathologies. Although moderate membrane thickening is accepted as normal, > 2 mm thickening is considered a symptom of sinusitis [24]. Sinus membrane thickening in periapical lesions or endodontics infections was investigated and it was reported the percentages ranged from 35.8 to 89.4% [2, 4, 5, 8, 15, 16] The difference in these percentages may be due to the fact that the accepted value for membrane thickening varies between studies. In the literature, the cutoff value for SMT is in the range of 1 –6 mm [5, 6, 8]. While most studies reported a value greater than 2 mm as membrane thickening [2, 4, 9, 14], others accepted this value as 3 mm [23]. A previous study accepted the cutoff value for the SMT is 1 mm, and in that study, quite high values were reported as 76.1% and 91.4% for adequate or inadequate root canal fillings, respectively, or 67.7% and 89.4% in the absence and presence of periapical lesions, respectively [8]. Prothikhun et al. [16] also accepted the cutoff value as 1 mm and stated a 35.8% thickening in the presence of canal filling, regardless of its quality, and a 35.9% thickening in the periapical lesion. In our study, the cutoff value was 2 mm and the thickening in the sinus membrane was detected at the rate of 33.45% in the teeth with adequate filling quality and whose apical foramen opened into the sinus cavity. Since there is no clear consensus regarding the thickening value of SMT, it becomes difficult to compare the results of the studies. In this study, in addition to percentages, measurement values in mm were also reported.

Previous studies have measured the bone distance between the periapical lesion and the sinus cavity and examined how much the proximity of the periapical lesion to the cavity affects it [2]. Other studies have examined the distance between the root apex and the sinus cavity [25, 26]. In a study examining the effect of endodontically treated teeth on SMT, the root position classification of Chan et al. [27] was used and the roots were classified as not in contact with the sinus, in contact, and penetrated. In some anatomical structures, although the roots are in contact with the sinus from their apicolateral surfaces, their apical foramen are included in the bone borders [28]. This creates an inaccuracy during the examination of the influence of endodontic therapy. In this study, healthy endodontically treated teeth whose apical foramina were open to the sinus cavity were evaluated. If there is healthy bone between the apex of a healthy tooth and the sinus cavity, it becomes difficult to detect the micro-effect of root canal treatment performed under adequate conditions and properly on the sinus. Therefore, teeth whose apical foramen were not associated with the sinus cavity were excluded from the study.

A successful root canal treatment maintains the health of the periodontal ligament beyond the apical foramen and the bone in the periapical region. However, the effect of root canal treatment applied to a tooth whose roots open into the sinus cavity on the sinus membrane and sinus cavity may be different from its effect on the periodontal ligament and periapical bone tissue, since the characteristics of the tissues are different. In this study, it was shown that the protrusion of the endodontically-treated root into the sinus caused a minimal increase in SMT. This can be related to the increased relationship between the root canal system with the maxillary sinus not only through the apical foramen but also through the dentinal tubules and lateral canals. Nevertheless, more cross-sectional studies are needed to confirm this result.

According to the results of our study, when root canal treatment is performed to maxillary molars extending into the maxillary sinus, it is recommended that microbial control in the entire root canal system should be maximized in order to minimize possible sinus membrane irritations. Besides, especially in the complex canal morphology in the apical region, cleaning the root canal from organic and inorganic residues by activating irrigation solutions and adequate mechanical instrumentation, and performing a canal filling with high biocompatibility is quite important.

In this study, the effect of the time periods between 12 and 24 months of root canal treatment on sinus membrane thickness was analyzed. It was observed that endodontic treatment performed over a period of 1–2 years had no effect on the sinus membrane thickness. In 2021, within the scope of the clinical practice guideline, a minimum follow-up period of 1 year is recommended for evidence of the emergence of apical radiolucency, as a consensus-based development report in measuring the outcomes of endodontic treatment [29]. In this study, images with 1–2 years of endodontic treatment were included.

One of the limitations of the study is the sinus examination was not performed by an ear-nose-throat specialist to detect the presence of any sinus pathology. The data is based on the patient’s medical history. In previous studies, the effect of endodontically treated teeth without pathology on sinus membrane thickness has not been examined. It is clear that an endodontic periapical pathology will affect the surrounding tissues such as the sinus [8, 15, 16]. The strength of this study is that it examined the effect of a healthy endodontic treatment on sinus membrane thickness. However, although the endodontic treatments were performed by one general dentist in the private dental clinic where the data were obtained from which the CBCT images were evaluated retrospectively, it would not be correct to say that the procedures of the endodontic treatments were fully standardized due to the nature of the retrospective study. A prospective cohort study should be designed to deeply understand the effect of the parameters that were analyzed in this study.

## Conclusion

Within the limitations of the study, it was concluded that the sinus membrane thickens in the presence of endodontically treated teeth whose apical foramen protruded into the sinus cavity, with a rate of 33.45%. In case of root protrusion, the clinician should be careful to prevent a possible sinus reaction due to the increased interaction of the root canal system and the sinus membrane via the lateral canals and dentinal tubules. Sinus membrane thickness did not differ between the first and second molars or genders.

## Data Availability

No datasets were generated or analysed during the current study.

## Declarations

### Additional information

A pilot form of the study was orally presented at the 9th International Endodontic Symposium in Türkiye.
